# Introduction of home-based mobile specialist psychiatric nursing teams: Experiences of mental healthcare professionals

**DOI:** 10.1186/s13033-026-00733-w

**Published:** 2026-09-10

**Authors:** Susann Arnell, Agneta Schröder, Ylva Nilsagård, Linda Ryen, Maria Fogelkvist

**Affiliations:** 1 University Health Care Research Center, Faculty of Medicine and Health, Örebro University, Örebro, Sweden; 2 Department of Health Science, Faculty of Health, Care and Nursing, Norwegian University of Science and Technology (NTNU), Gjövik, Norway; 3 University Health Care Research Center, Örebro University Hospital, Örebro, SE-701 85 Sweden

**Keywords:** Healthcare professionals, Home treatment, Psychiatric nursing team, Mobile team

## Abstract

**Background:**

The number of adults in need of psychiatric care is increasing in Sweden. To meet this need, new working methods are implemented. Home-based mobile specialist psychiatric nursing teams (SPOT) are being introduced to function as a complement to outpatient care and inpatient care, but still little is known about how healthcare professionals experience such efforts. The purpose of this study was thus to capture mental healthcare professionals’ experiences of the early phase of the introduction of SPOT.

**Methods:**

Focus group discussions and semi-structured interviews were conducted with 11 mental healthcare professionals, including nurses, psychiatric aides working within psychiatric care and on the SPOT team and psychiatrists who had a consultative role in SPOT. The data was inductively analysed through reflexive thematic analysis.

**Results:**

The discussions and interviews revolved around three themes: *The phases and conditions of the change*, *Opportunities and challenges in the new form of intermediate care* and *Care in the patient’s home for patient empowerment and engagement*. Different perspectives on the new form of care emerged and many were positive about patients’ increased engagement in care when it was offered in the home environment. However, it was also emphasised that the need for different forms of care remains and that a new level of care should not exclude other forms of care. The results further showed a need to engage and offer continuous support to healthcare professionals at different levels when new working methods are introduced. Specifically, support from managers and increased competence and skills development in performing new work tasks were requested.

**Conclusions:**

Providing care in the home environment through SPOT interventions can help increase patients’ engagement in their own care. Preparing and equipping healthcare professionals at all levels and offering them knowledge support and influence in the introduction of new forms of care is important to reduce the vulnerability that professionals may initially experience in change processes and new ways of working.

**Supplementary Information:**

The online version contains supplementary material available at https://doi.org/10.1186/s13033-026-00733-w.

## Introduction

The number of adults in need of psychiatric care in Sweden is increasing, but the number of inpatient care beds is on the decrease [1]. This development is also seen internationally [2]. Psychiatric care is responsible for preventing and treating mental disorders and for improving care to achieve good quality care for all patients [3]. Good quality in healthcare entails that the provided care is, among other things, effective and safe [4]. Receiving good quality care is considered a right for all patients [5], while staff have a responsibility to provide the best possible quality of care [6]. Moreover, psychiatric care should be person-centred, which can be a challenge in situations where patients present with acute illnesses that warrant inpatient care or other restrictive measures, with or without the patient’s consent [7]. An alternative to inpatient care is home treatment, which has been proposed to offer a model of care that is more person-centred [8].

In Sweden, there is an ongoing shift from reactive to more proactive care, going from a system built around diseases and institutions to a system designed for people. With this reform, patients are no longer seen as passive receivers of healthcare; instead, they are invited as co-producers, where their abilities and needs are central [9]. As a result, mobile psychiatric teams are being introduced in many Swedish regions, and they are supposed to function as a complement to care at other levels (outpatient care and inpatient care). Offering interventions from mobile teams means that care is moved to the patient’s home, which assumably affects the patients and their next of kin but also the healthcare professionals.

Results from a scoping review showed that the introduction of mobile teams can reduce the number of admissions to inpatient care, and that patients were more satisfied with home treatment in some of the included studies [10]. However, there is great variation in how mobile teams within psychiatric care are organised and what their assignments consist of [11]. One model that has received great research attention is that of the crisis resolution team offered to patients experiencing an acute and severe mental health crisis [12, 13]. Crisis resolution teams are often characterised by offering easily accessible interventions, which are short-term, intensive and offered in the patient’s home regardless of the time of day [14]. This is seen as a promising alternative to inpatient care, but it is still unclear what elements of such interventions are effective [10].

In Region Örebro County, Sweden, the executive management decided in the fall of 2023 to close one of its four inpatient wards and to launch a new form of care providing home-based specialist psychiatric care through nursing teams instead. This initiative has come to be known as SPOT. The SPOT team offers person-centred psychiatric care in the patient’s home environment. The goal is to prevent the need for hospital care by offering care close to the patient at an early stage of disease deterioration and in connection with discharge from 24-hour care. However, it is still unexplored how mental healthcare professionals in a Swedish context perceive the introduction of these mobile psychiatric teams. The transition from inpatient care to specialised mobile teams thus needs to be studied from the perspective of mental healthcare professionals. Although studies show that nurses are more satisfied with providing care at home [15], more studies are needed that attend to the perspectives of professionals on different types of mobile psychiatric teams. This can contribute to a greater knowledge base for the continued development of mobile teams in a Swedish context. The aim of this study is to capture and describe mental healthcare professionals’ experiences of the early phase of the introduction of SPOT.

## Method

### Design

This study uses data from focus group discussions and semi-structured interviews with mental healthcare professionals with experience in psychiatric care and connected to the introduction of a new form of care through the SPOT team.

### Setting

In the region of the study, psychiatric care is organised into outpatient and inpatient care. A psychiatric emergency department offers assessments of need for inpatient care 24 h a day, 7 days a week. Further, if a patient visiting an outpatient clinic is assessed as needing inpatient care, a psychiatrist is consulted, either by seeing the patient or by telephone consultation with the primary caregiver. SPOT is intended to provide an intermediate psychiatric model of care that can be initiated from all these instances. A statement of work to introduce the SPOT model was formulated in the summer of 2023, stating that a functioning SPOT team should be in place by the end of 2023. The new SPOT team was developed mainly by gathering information from other regions in Sweden that had already integrated home treatment as an alternative in psychiatric care, and it was also customised based on the availability of staff from the closed ward. Mental healthcare professionals employed at the ward included nurses, psychiatric aides and psychiatrists. Contact with SPOT is offered to patients who show a deterioration in symptoms, patients who are assessed as not warranting inpatient care and patients discharged from inpatient care. Exclusion criteria for interventions via SPOT, according to the initial local guidelines, were substance use disorders and severe intellectual disabilities. Contact with the team, via home visits or telephone, is offered 16 h a day, and care is provided in collaboration with the patient and their next of kin in a safe and supportive environment.

### Participants

All mental healthcare professionals (nurses and psychiatric aides) working on the SPOT team and the psychiatrists working in psychiatric care who had a consultative role in SPOT were invited to participate (*n* = 16). Contact details for potential participants were obtained from the psychiatric care managers and study information was sent out by email. The participants were informed that their participation was voluntary. All participants gave written informed consent.

Twelve mental healthcare professionals agreed to participate in the study, of which one dropped out. To be able to transparently report the composition of the group of professionals, they were asked to answer questions regarding profession, years in profession and time on the SPOT-team (Table 1).

**Table 1:** Informant characteristics

Informant characteristics		
**Profession**		N (%)
	Nurse	2 (18%)
	Psychiatric aide	5 (46%)
	Medical doctor	4 (36%)
**Years in the profession**		Mean (SD):
		16.10 (11.15)
**Time on SPOT-team**		N (%)
	> 6 months	10 (91%)
	< 3 months	1 (9%)

### Data collection

The data were collected one year after the implementation of SPOT had started, when about 130 patients already had received care through SPOT. Focus group discussions and semi-structured interviews were chosen because they are useful methods to explore experiences and views among small groups of individuals who have some involvement in the topic under investigation [10, 11]. An interview guide was collaboratively developed by the research team with input from patient and next-of-kin representatives. The focus group discussions and interviews were based on six key questions, which were developed to capture the participants’ experiences of a new form of care through the SPOT specialist psychiatric nursing team (Appendix 1). The interview guide was used throughout the data collection, including prompts and probes to enable a detailed discussion of participant responses.

### Procedure

The focus groups were planned to include four informants each. However, as one informant dropped out and because of further difficulties in coordinating times for focus group discussions, data was collected in various ways, using focus groups and joint as well as individual interviews.

The final focus groups consisted of four participants in one group and three participants in the other group. Of the remaining participants, two were interviewed jointly and two individually.

During the focus group discussions, the first author (SA) had the role of moderator, leading the focus group discussions, and the third author (YN) had a role as an assistant moderator, occasionally asking follow-up questions to clarify the participants’ opinions. The discussions were carried out as a group conversation, using probes and follow-up questions to obtain rich descriptions and knowledge. The focus group discussions lasted about 90 min. To support the dependability of the study, an audit trail was produced where immediate reactions after focus group discussions were recorded by the participating researchers.

The two semi-structured individual interviews and one joint interview were conducted by the last author (MF) in collaboration with the second author (AS). The semi-structured interviews lasted between 41 and 58 min. All interviews and focus group discussions were audio recorded and transcribed verbatim. There were no dependent relationships between the participants and the researchers.

### Data analysis

The data was analysed through an inductive, semantic, reflexive thematic analysis in accordance with the outline provided by Braun and Clarke [16, 17]. This is to maintain an empirical and realistic approach where researchers stick to what is current, relevant and close to reality. Reflexive thematic analysis also captures the active role of the researcher and the researcher’s actions, assumptions, expectations, values and choices as well as the effect and influence they have on the research throughout the research process [17].

The transcripts were de-identified, and the interview recordings were listened to in order to ensure that the transcriptions were accurate, and any errors were corrected. The analysis then followed the recommended six phase iterative process of Reflexive Thematic Analysis [17]. The first phase included familiarization with the data, the transcripts were read several times by all authors to gain an overview of the content. Then, in the second phase, the members of the research team independently reviewed the transcripts and generated the initial codes. The transcripts were then coded in their entirety by SA and MF. In the third phase, the initial theme generation began by sorting similar codes into preliminary or initial themes. A series of tables containing key aspects were used to organise the data. Any discrepancies between codes or initial coding were resolved through discussion.

This iterative process resulted in provisional themes, which in the fourth phase were further refined and aggregated by content to produce the final themes. In the fifth phase, including defining and naming themes, the descriptively labelled themes were continuously compared with the data to ensure that the content could correctly be derived from the interviews and encompassed their content. In the final, sixth phase, the writing process was completed, and several quotes were chosen to illustrate the interviews and focus group discussions. Although the participants represented diverse categories of professionals, their small number would not allow an analysis by subcategory [18] and the statements of each individual participant were therefore considered personal reflections, not necessarily representing a particular profession. The NVivo 14 Software [19] was used in the thematic analysis.

### Researcher reflexivity

The research team comprised five individuals with backgrounds in psychology, psychiatric nursing, health economics and physiotherapy. To improve reflexivity, researcher triangulation was utilised, where each researcher’s interpretations and knowledge were considered in a collaborative way through a collective process within the research team [20, 21]. The impact of the research team’s knowledge and expertise in psychiatric care, quality of psychiatric care, management, integrated care and implementation science was continuously discussed throughout the different stages of the study. A reflexive journal was kept during data collection and analysis [17].

### Results

The results in this study build on healthcare professionals’ experiences of the early phase of the introduction of SPOT. The healthcare professionals rated their overall experience of SPOT as positive. Ten of the informants had more than 6 months experience in working on the SPOT-team, one had less than 3 months experience (Table 1).

The thematic analysis resulted in three themes: *The phases and conditions of the change*, *Opportunities and challenges in the new form of intermediate care* and *Care in the patient’s home for patient empowerment and engagement*.

#### The phases and conditions of the change 

Mental healthcare professionals concerned with and involved in the new SPOT team reflected on the need for organisational preparedness and the importance of being able to have influence during change processes. Communication and the timing of change work were considered important, as was the continued work on further developing the new form of care.

##### Implementing SPOT

The professionals in the study had varying knowledge of the background behind the change and varying understanding of the need for the change process that resulted in the introduction of SPOT as a new intermediate care unit. The doctors to a greater extent described various factors that led to the change process, where competence and staffing issues, such as difficulties in recruiting personnel, were highlighted as central aspects. The psychiatric nurses and aides instead described that the decision to introduce a new intermediate care unit was communicated shortly before the closure of the psychiatric inpatient ward where most of them had been employed. The timing and way in which this was communicated – during or just before their vacation – caused upset feelings. It was difficult for the healthcare professionals to absorb the information, which led to many questions initially remaining unanswered:

“*It was a hasty decision … to close the ward*,* the grief we went through … To start something new and turn things around and get into the positive mindset of ‘Yes but we are part of shaping something new’ … it was … messy*” (F 2:4).

The healthcare professionals, especially the psychiatric aides, described a sense of worry and uncertainty:

“*there was a lot of worry during the holidays when you didn’t know if you would get a position* [job] *or not*” (F 2:2).

The mental healthcare professionals who were hired to work on the SPOT team described that much was unclear and incompletely prepared before the start of the new form of care, but that they together were able to shape the team’s way of working. The SPOT team made study visits to other similar healthcare units and received training related to the new assignment, but many questions and challenges remained. During the initial planning phase of the SPOT team’s work, employees felt supported by the project group and felt that they had influence, with their voices being listened to, which they considered important.

##### Evaluating and scaling up

The participants described that at the time of the interviews, the SPOT team’s activities had not yet been evaluated, since only a year had passed since its introduction. So far, about 130 patients had received interventions from the team. According to the participants, the team’s working methods had not yet found their optimal form, and it was questioned whether the efforts could be evaluated by assessing the patients’ satisfaction only:

*“… how to evaluate? … it’s very easy to show that everyone is satisfied*,* the staff feels that they are doing meaningful work*,* the patients are satisfied*,* the next of kin are satisfied … but … have you really made any difference?”* (I 3).

Despite concerns among the healthcare professionals, they described that initiatives to spread and scale up this form of care had already begun. This worried some of the participants, who thought it was going too fast:

“*I think it’s good that we’re testing*,* but I think it’s going a little too fast in psychiatry that there’s some kind of ‘SPOT mania’ going on with all the* [local sections] *that want to start a SPOT team. So even though I’m very positive myself*,* I don’t have the facts to show that it works and that it will in any way meet the goal. You can’t see that because it’s too early and these numbers*,* you can’t*,* you don’t know*” (I 1:1).

#### Opportunities and challenges in the new form of intermediate care

SPOT faced challenges when communicating its mission to other units within the healthcare system. The introduction of this new form of care led to changes in the professionals’ roles and responsibilities, which led to discussions about competence issues but also highlighted the vulnerability that the healthcare professionals experienced.

##### Varying views and knowledge

SPOT was described by some of the participants as a complement, a new form of intermediate care enabling smooth transitions between inpatient and outpatient care. Another purpose of SPOT that was described was to relieve psychiatric emergency departments by having fewer patients seek care and for SPOT to be able to identify and meet the needs of these patients. Others described SPOT as a replacement and linked it to the time when the change process began:

“*I think something negative was that the SPOT team started at the same time as the ward was shut down … So everyone thought*,* ‘Okay*,* now we’re going to have SPOT instead of a closed psychiatric ward.’ It was unfortunate that it happened like this*,* you could have both and then everyone would think it was only positive*” (I 1:1).

The somewhat unclear assignment given to the healthcare professionals also led to SPOT being perceived in varied ways, as an intervention that was initiated at the expense of other interventions within psychiatric care:

“*My biggest concern was that it was introduced as a replacement for reduced inpatient care*,* access to inpatient care*,* which I … I think we have reached the limit for the number of inpatient beds* [i.e. the lowest number] *where we can’t compensate with SPOT-like solutions*” (I 3).

Some described SPOT as.

“*a kind of luxury* [service]… *if it’s patient-safe and quality isn’t affected …”* (I 2),

which should not be seen as a substitute for well-functioning psychiatric outpatient or inpatient care. One concern that was discussed was that resources would be taken from inpatient and outpatient care, which already are extremely strained, to build up new SPOT teams:

“*then we will build such a luxurious effort at the expense of … having outpatient care to turn to and having inpatient care when there is a crisis*” (I 3).

The healthcare professionals on the SPOT team feared that the unclear mission and a lack of knowledge about SPOT’s assignment risked leading to the team’s expertise not being utilised, and that there was a risk that the “S” (specialised) in SPOT would be lost. This could lead to the “wrong” patients being referred to the SPOT team, which in turn leads to SPOT.

*“traveling around as some kind of nursing team and housing support … that would be a shame*,* because then … then the care team for our patients who are sick has really disappeared*” (F 2:4).

##### The changed way of working

The healthcare professionals experienced that SPOT brought with it other tasks:

“*it is quite a big change in tasks*,* there is more focus on the caring conversation*,* making your own assessments*,* making plans together with patients*,* which perhaps means a little more demands on yourself as an employee*” (I 1).

The changed way of working also brought with it other requirements for documentation. A new way of documenting in patients’ records created frustration among some professionals:

“*Documentation is much more complicated now*,* it’s the worst part of the job*” (F 2:2).

The new way of documenting was perceived as time-consuming, which meant that less time could be allocated to working directly with patients:

“*sometimes the administrative work takes as much time as the* [home] *visit*” (F 2:2).

The nursing professionals described certain aspects of their work as difficult and challenging. The psychiatric aides were not used to making independent assessments of the patients’ condition and care needs, which formed the basis for their continued care. They felt that they lacked sufficient training and were not fully prepared for this task. The level of responsibility in relation to competence was questioned, which was also addressed during the focus group discussion:

“*Do you have the feeling that you have that competence or that you have been given a responsibility that you are not really prepared or able to take on*?” (F 1:2).

The new tasks were described as more demanding in terms of responsibility and by some even as feeling.

“*burdened with responsibility*”, “*that responsibility is also more difficult because they are not here* [at the ward], *how can I assess in the same way as I could with someone who is here*,* … but now maybe they are … 70 kilometres away*,* how can I do this on the phone*,* how can I get my medical standby to think the same thing as I am experiencing*” (F 2:3).

The new responsibility was experienced as particularly heavy when it could not be handed over to another healthcare professional and when the patient was left at home instead. At the same time, the healthcare professionals described that their confidence in their own ability to make independent assessments increased over time and they felt that they had a different role, that they were listened to in a different way:

*“… I think we make assessments in a completely different way*,* I think you have a completely different dialogue with the doctor … they listen more to how we have assessed*,* how we see the situation*, etc.,* so I think that the dialogue has probably become better*” (F 1:1).

##### The vulnerability of the healthcare professionals

The new way of working brought with it several new challenges for healthcare professionals, who described a vulnerability on several different levels:

“*you didn’t get any preparation time*,* you didn’t get any extra training*,* you didn’t get any support or guidance for dealing with all these emotions either*” (F 1:2).

Although several healthcare professionals appreciated meeting patients in their home environment, they also described a vulnerability linked to the work environment:

“*you’re exposed in a completely different way*,* when you go to someone’s house. There are two of you and you go there by car*,* and you don’t really know what you’re going to meet*” (F 1:1).

They considered it important to have previous knowledge of the patients, but meeting several new patients contributed to this feeling of vulnerability instead. They further described that they did not always know who they would meet during the home visits:

“*and is it even the patient you are meeting … or is it the patient’s cousin*,* neighbour*” (F 1:2).

Even meetings with the patient’s next of kin could be experienced as challenging:

“*you are held accountable for all the shortcomings of psychiatry at all levels*” (F 1:2).

Other aspects discussed were the vulnerability healthcare professionals experienced in connection to deficiencies in staffing, skills and support. A shortage of certain professional categories contributed to the work being conducted with different staffing than initially planned. Consultations with doctors were experienced as troublesome, and healthcare professionals described feeling questioned in situations where they asked for support in their assessments. This highlighted the need for increased competence:

“*you have to invest more in internal training* … *so that you feel competent and you feel comfortable and you feel safe when you go out and make your assessments and when you train new staff*,* so that you really feel that you can live up to that name*” (F 1:2).

The lack of support in patient matters and the lack of targeted support for professionals to manage emotions was discussed:

“*When you go to people’s homes*,* it brings up so many more things than when you’re on a ward where you hand over to someone else anyway*,* then the next shift comes and takes over*,* it’s a huge*,* huge change*,* a huge difference*” (F 1:2).

The perceived lack of support from the management level became clear and the healthcare professionals were referred to processing events/emotions through peer support within the team instead.

#### Care in the patient’s home for patient empowerment and engagement

Despite the challenges and questioning linked to the introduction of the new form of care, the healthcare professionals deemed that SPOT was generally good for the patients. Through SPOT, adapted interventions were made available for a patient group whose needs lie between outpatient and inpatient care. This form of care increased the healthcare professionals’ insight into the patients’ life and care needs, and it also enabled next of kin to be involved in the care. Care and treatment in the patient’s home resulted in a changed balance of power between patient and healthcare professional, which made healthcare professionals perceive the patients as more involved and responsible.

##### Better care for the "right" patient

A general perception among the healthcare professionals was that SPOT can offer accessible and tailored care for the right patient group at the right time. Other aspects that were described were that interventions could be initiated quickly and that healthcare had the opportunity to reach out and have contact with new patient groups that had previously been difficult to reach, such as patients who were hesitant to receive help via inpatient care in psychiatry. According to the professionals, patients described this increased accessibility as reassuring. The importance of the “right timing” was explained in terms of being able to offer interventions at an earlier stage in the disease process (before the need for care in the ward) and that any deterioration could be identified earlier. The ambition to strengthen the patients’ healthy side through care and treatment at home was described, while the importance of ensuring that the right group of patients had contact with SPOT was emphasised.

##### Meeting the patients in their home environment

A contribution to better care was that healthcare professionals gained a better insight into patients’ lives when visiting them at home. The healthcare professionals’ views of the patient could change:

“*Huh*,* is this the same person? Suddenly this became a different person in front of us*,* like you became … well I … had tears in my eyes when we left because I felt like this*,* ‘How nice to see the person*,* not the patient*’” (F 2:4).

Home care also meant that other needs were recognised and collaboration with other actors could be initiated if necessary:

“*You get a completely different unique insight into the person’s situation*,* they may seek treatment for anxiety and then you go home and then you get a full understanding of what all that anxiety is*,* or a person’s functional ability*,* you can never see that during a visit* [to the care unit], *but when we are at home*,* then you can see what their functional ability is or is not*”(F 1:2).

The conversations with the patients also changed and had a different character:

“*It’s a different meeting with the patient*,* it becomes more of a conversation*,* now we’re going to sit down and we’re going to talk …*” (F 2:3).

This meant focusing more on the caring conversation, which placed different demands on the healthcare professionals:

“*On the ward it was a bit more like you could see each other several times and then maybe you’d sit down and then you’d talk a bit about what was in the room*,* it’s a slightly different approach to the conversation*,* … it’s been a change for me in how I should hold the conversation to get the right things out in the conversation*” (F 2:3).

Home care also affected the balance of power between patient and healthcare professional compared to care provided on a ward:

C*oming to the patient’s home*,* then we are guests in their home*,* here* [on the ward] *they were guests in ours. … that is*,* in inpatient care. Just because you have hospital clothes*,* you have a different power than what you have at someone’s home. If I come to your home and knock on your door and sit on your couch*,* there they are the ones who own the situation in a completely different way … I have experienced that as a big difference.* (F 2:3)

The professionals also described that.

“*It feels less threatening* [to meet patients in a home environment] *than on the ward*,* as they are often more relaxed at home*” (F 2:2).

The healthcare professionals also felt that home care contributed to increased participation among patients, which also led to increased personal responsibility, which was something positive.

##### The next of kin''s involvement in care

The healthcare professionals experienced that the involvement of the patients’ next of kin in their care and treatment changed when it was offered through home visits by the SPOT team. According to the healthcare staff, next of kin could be helpful in describing the patient’s situation, especially if the patients themselves had difficulty communicating and answering questions or lacked insight into the disease. At the same time, the involvement of next of kin was described with some ambivalence, for example when they “take over” the conversation and the patient’s own voice is not heard. The healthcare staff also described that it could be challenging to have to meet the next of kin’s expectations of care and of the new SPOT team. Having the patient receive care and treatment at home instead of being admitted to an inpatient psychiatric ward can also mean that their next of kin do not receive the relief they may need. Here, the healthcare staff highlighted the need for next of kin to also be informed about the SPOT team’s mission:

*“… it is important … that next of kin also have information about what SPOT’s interventions are*” (F 1:1).

## Discussion

The aim of the present study was to describe professionals’ experiences of the introduction of a new specialist psychiatric nursing team (SPOT) in the northern part of Region Örebro County, Sweden. This qualitative study revealed different perspectives on SPOT as a new form of mental health care, including different opportunities and challenges related to its implementation.

Opportunities and challenges were described somewhat differently between mental healthcare professionals who were part of the SPOT team and doctors who primarily had a consultative role. In this context, it was obvious that psychiatric nurses and aides were directly affected in their employment while doctors were more affected in their duties, where SPOT interventions constituted a limited part. The background of why a new way of working was introduced was described more clearly by the doctors. The nurses and psychiatric aides instead described the different implementation phases in more detail. The profession thus seemed to influence how SPOT was viewed.

An important finding in this study was the vulnerability described by healthcare professionals during the initial implementation phase. This vulnerability was linked to the timing of the change and the perceived lack of support from management, which became apparent after the additional support that was available during the first month was removed. Previous research shows that the manager’s commitment and active role in a change process is related to intervention outcomes and employees’ well-being [22]. Given that mental health professionals’ well-being affects the quality of care they provide to patients, managers play an important role in supporting their professionals [23]. In addition, support from managers may also reduce burnout among professionals [24].

Lack of training and poor awareness of new interventions are considered key barriers in their implementation [25]. This also became evident in this study when new tasks, which some of the professionals had no experience in performing, contributed to the feeling of vulnerability. There were variations in the level of knowledge about the new SPOT team, its purpose and which patients could be referred to it, and also in how the assignment was interpreted. Ambiguity related to SPOT’s mission was evident, with the psychiatric nurses and aides on the team fearing that it risked becoming a slop bucket, especially because the criteria for which patients should have interventions via SPOT were not always followed. The doctors instead feared that resources were not being used or allocated optimally and that SPOT risked being considered a “luxury product”. Time will tell whether or not these concerns are dealt with and thereby avoided. Professionals with greater overall responsibility assessed SPOT from an organisational perspective, while psychiatric nurses and aides instead were able to describe how the change affected patients in their everyday lives in more detail. The pace at which SPOT was introduced and scaled up was, however, questioned by the healthcare professionals and they addressed the need for a proper follow-up. Another concern was linked to resources, especially personnel shortages, which were described as a recurring problem. Various needs thus need to be considered in the implementation of patient-focused interventions – needs linked to the system, to the staff and to the intervention [25] – and adequate resources are needed to cover for “critical ingredients to successful implementation”, including support and promotion of team engagement and ownership [26].

Despite all the challenges and initial difficulties, the healthcare professionals felt that SPOT made an important contribution to psychiatric care, and they described their overall experience of SPOT as positive. The nursing staff believed that patient participation was improved when care and treatment were carried out at home. Patients and their next of kin were considered more involved and were perceived to have greater influence and responsibility compared to when care was received on a ward. The opportunity to have influence was considered important, regarding both patients’ influence on the care offered and professionals’ influence on the care provided. This is in line with the intention of the reform – to see patients as co-producers instead of passive receivers. Previous research shows that influence or participation is linked to perceived quality of care, but both professionals and patients generally perceive this to be low within psychiatric care [27, 28]. However, previous studies have shown that opportunities for participation seem to be a well-known dilemma in psychiatric care [29–31]. Our results suggest that an approach that offers care in a home environment may be a path to increased engagement and personal responsibility among patients in psychiatric healthcare. Given the similarities across regional healthcare structures in Sweden, it is conceivable that a SPOT-like form of care could function similarly in other regions. However, many healthcare professionals remained sceptical about the impact of the SPOT interventions, given that so few patients had received care from the team at the time of data collection and the evaluation methods were called into question. Therefore, as home-based mobile specialist psychiatric nursing teams are now being introduced in several Swedish regions, it is important to conduct further research to investigate the applicability and effectiveness of this form of care in different settings.

In a recent literature review, criteria for how the effectiveness of psychiatric home-care mobile teams can be evaluated were examined [32]. The review reveals that the evaluation criteria are diverse and not yet standardised. To ensure both effective and high quality psychiatric care, it is important to assess and incorporate patients’ and professionals’ perspectives on the care received and delivered [33].

### Methodological considerations

The choice of focus group discussions and semi-structured interviews as data collection methods enabled the views of healthcare professionals, both women and men of different ages, with different backgrounds and professional roles, to be considered. An experience-based design method and involving patients and next-of-kin can contribute necessary knowledge in research and all development and evaluation work [34]. The input from the patient and next-of-kin representatives in this study was valuable when designing the interview guide, for example regarding collaboration with different actors. The study design had to be compatible with the implementation of the SPOT project, which already had been conducted. While focus group discussions make it possible to capture several perspectives and gain a picture of the shared understandings and the interplay between the SPOT team members [35], individual interviews allow for in-depth questions to be asked. Combining these two methods may have increased data richness. To further ensure the credibility, variety and richness of the data, healthcare professionals with different professional education and experience were included in the study. Various other measures were also used to enhance the trustworthiness of the study. The use of member-checking as a final step of the focus group discussions and the use of quotes were measures to achieve credibility. To achieve dependability and confirmability, we documented each step, strived for stability in data collection (which was performed over a narrow period of time) and kept an audit trail to enhance transparency [36].

The study also has some limitations, some of which are related to the short period of time the SPOT team had been operating. At the time of data collection, relatively few patients had received interventions via SPOT. To better evaluate professionals’ experiences of a new form of care, follow-up is needed also when a longer period has passed since the intervention was implemented, preferably in a systematic and continuous manner.

### Implications for practice and research

Evaluating professionals’ experiences in the early phase after introducing a new form of care in psychiatric care contributes to improving treatment and nursing interventions for patients. It also clarifies professionals’ need for support in implementing new interventions and the need for long-term strategic planning to manage personnel shortages. Challenges experienced initially can clarify needs related to skills and training. Needs may change over time and look different at a later stage. Initiatives that enable engagement and let employee voices be heard are crucial for employee well-being [37], and evaluating initiatives and, in this context, considering professionals’ needs in relation to system and organisational needs should therefore be an ongoing process. In addition to the professionals’ perspectives, studies are needed that illuminate the perspectives of patients and relatives, which are crucial when new forms of care are introduced. Two other studies are ongoing within the project, one study with a patient and family perspective to shed light on their experiences and another study focusing on the introduction of SPOT teams and effects on resources.

## Conclusions

In summary, this qualitative study highlights the importance of involving and preparing mental healthcare professionals, at different levels within the organisation, when introducing a new form of care. The results show that the introduction of a specialised SPOT team brought both opportunities and challenges. Providing care through SPOT interventions can help increase patients’ engagement in their own care and thereby promote the development of a more patient-centred approach. However, perspectives from different professional groups also helped to clarify different views on the advantages and disadvantages and possible risks associated with the introduction of a new form of care, highlighting the need for continuous follow-up. The importance of giving influence, offering knowledge support, and preparing and equipping healthcare professionals at all levels cannot be understated when it comes to minimising the vulnerability that professionals may initially experience in a change process.

## Supplementary Information

Supplementary Material 1

## Data Availability

The datasets generated and analysed during the current study are not publicly available due to considerations of confidentiality. Restrictions apply to the availability of these data, which were used under license for the current study. Anonymized, code level data (Swedish only) are available from the corresponding author on reasonable request.
