# Supplementary material for: Introduction of home-based mobile specialist psychiatric nursing teams: Experiences of mental healthcare professionals
Source: Int J Ment Health Syst. 2026 Sep 10;20:30. doi: 10.1186/s13033-026-00733-w (PMC13570513; doi:10.1186/s13033-026-00733-w)
Supplement: Supplementary file 1 [file 13033_2026_733_MOESM1_ESM.pdf]

Appendix 1

Interview guide

The opening question:
How have you experienced the introduction of the SPOT specialist psychiatric nursing team?

Six key questions/topics covering experiences:
Please, tell us about…

- **The changes in the form of care:**
  Possible prompts and probes:
  How did you find out about setting up mobile teams?
  What did you think then?
  How long have you worked in a mobile team?
  How would you describe the difference compared to working in 24-hour care?
  What do you think now: advantages/disadvantages/challenges/opportunities?

- **Providing care at home:**
  Possible prompts and probes:
  Being mobile team/traveling to provide care?
  Being in someone's home?
  Caring with next of kin around?
  Not having more colleagues nearby?
  Security/insecurity?
  The professional role – is it changing?

- **Differences in care?**
  Possible prompts and probes:
  Is it any different compared to care in a 24-hour care facility – content, dose?
  Does it affect documentation, establishing a care plan?
  Thoughts about the impact on patient safety, keeping track of medication?
  Thoughts about the impact on quality?
  Distribution of responsibilities?

- **Collaboration with other actors in society?**
  Possible prompts and probes:

  Collaboration with the care recipient/ with relatives / within the team / with municipal care and welfare?

- **Effects**
  Possible prompts and probes:
    What impact do you think home-based care has on the care recipient—can you give examples?
    Do you see any difference regarding the time it takes to improve/ the degree of improvement compared to 24-hour care?
    What are your thoughts on how providing care in the home affects the care recipient's next of kin?

- **Going forward**
  Possible prompts and probes:
    How would you like to see mobile team-based care develop?
    What would you need to achieve that?
    What risks or opportunities do you foresee?
